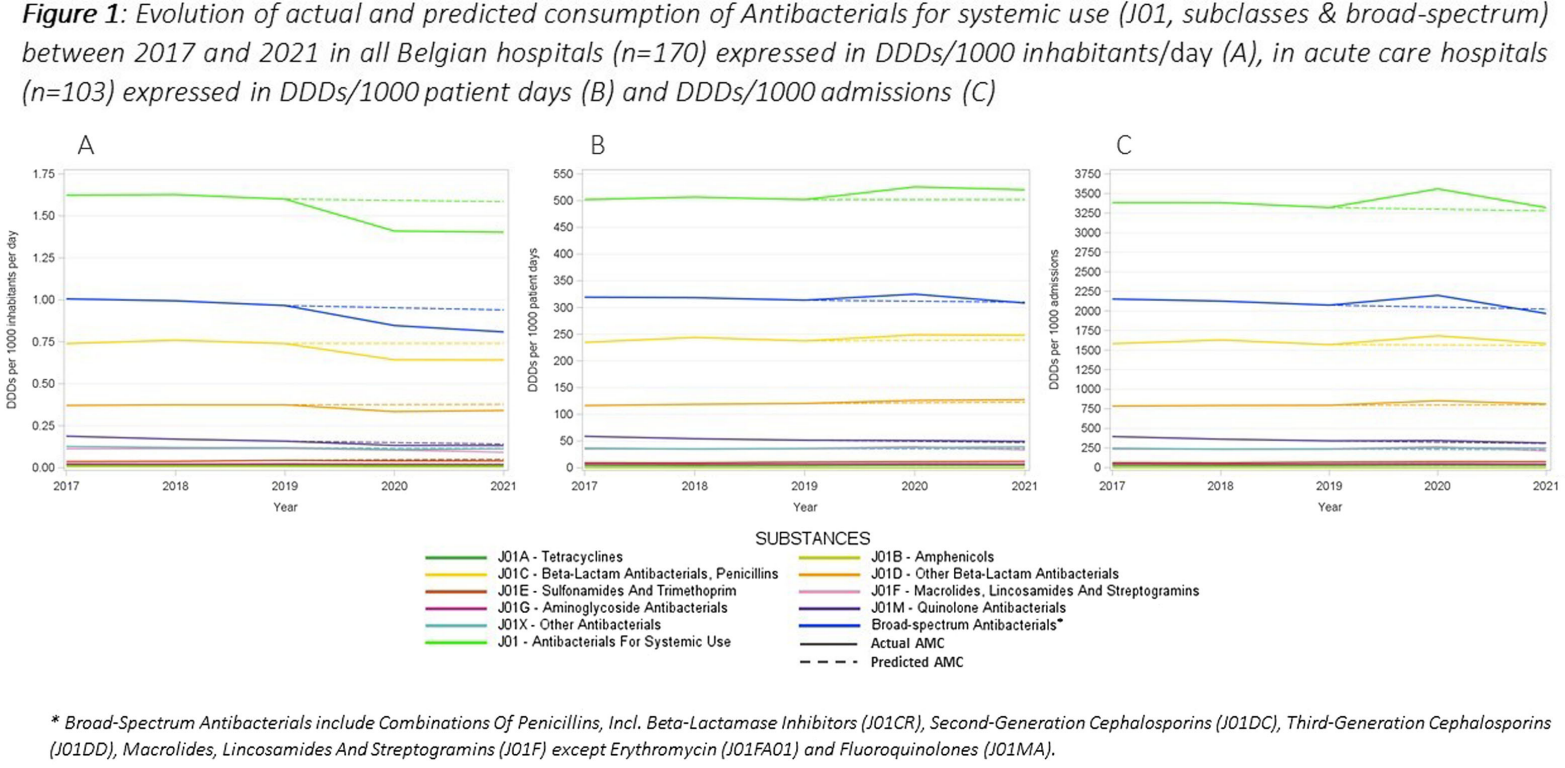# Trends in Hospital Antibacterial Consumption in Belgium (2017-2021): Evaluating the Impact of the COVID-19 Pandemic

**DOI:** 10.1017/ash.2024.326

**Published:** 2024-09-16

**Authors:** Laura Bonacini, Elena Damian, Boudewijn Catry, Lucy Catteau

**Affiliations:** Department of Epidemiology and public health, Sciensano, Brussels, Belgium; Sciensano

## Abstract

This study aimed to evaluate the impact of the COVID-19 pandemic on antimicrobial consumption (AMC) in Belgian hospitals from 2017 to 2021, using data from the European Surveillance of Antimicrobial Consumption Network (ESAC-Net) and the Belgian Hospitals Surveillance of Antimicrobial Consumption (BeH-SAC). Antimicrobial volume was quantified in Defined Daily Doses (DDDs), and AMC was expressed in DDDs/1000 inhabitants/day (DIDs), DDDs/1000 patient days and DDDs/1000 admissions. Linear regressions were employed to analyze 5-year trends for the ATC J01 group, at the ATC-3 level and for broad-spectrum antimicrobials. Broad-spectrum antibiotics included combinations of penicillins, incl. beta-lactamase inhibitors (J01CR), second-generation cephalosporins (J01DC), third-generation cephalosporins (J01DD), macrolides, lincosamides and streptogramins (J01F, excluding erythromycin J01FA01), and fluoroquinolones (J01MA). The compound annual growth rate (CAGR) calculated for the years preceding the pandemic was used to forecast 2020 and 2021 AMC, enabling a comparison with the actual use. Hospital AMC measured as DIDs decreased by 12% from 2019 to 2020. In contrast, when expressed as DDDs/1000 patient days and DDDs/1000 admissions, a 5% and 7% increase was observed, respectively. Antibacterials for systemic use (J01) showed a significant decrease over the 5 years only when expressed in DIDs. Notable trends included a negative trend for quinolone antibacterials (J01M) when expressed in the three incidence units, as for amphenicols (J01B) when using hospital denominators only. Positive trends were observed for sulfonamides and trimethoprim (J01E) using hospital denominators and for other beta-lactam antibacterials (J01D) with the ‘patient days’ denominator. While the consumption of all J01 antimicrobial subclasses deviated negatively from predicted use both in 2020 and 2021 when expressed in DIDs, positive deviations were recorded using hospital denominators, except for macrolides (J01F). The use of broad-spectrum antimicrobials showed a notable decrease between 2017 and 2021 when expressed in DIDs. However, when using hospital denominators, the observed use of broad-spectrum antimicrobials exceeded the forecasted values in 2020, to regress below the forecasted levels in 2021 (Figure 1). Contrary to results obtained using the widely applied country’s population as the denominator, a notable surge in AMC, particularly for broad-spectrum antimicrobials, was observed in 2020 when using hospital-specific denominators. This increase coincided with the onset of the COVID-19 crisis. These findings emphasize the need for a national hospital surveillance system that uses denominators that accurately represent the specific population being monitored. Implementing robust hospital-specific surveillance mechanisms would improve the precision of evaluations and facilitate targeted interventions aimed at optimizing antimicrobial utilization.

**Figure:**